# Five-year results after total knee arthroplasty in lymphoedema and lipoedema: encouraging functional and clinical outcomes and low rates of infection

**DOI:** 10.1007/s00264-022-05575-y

**Published:** 2022-09-08

**Authors:** Luke Granger, Scott M. Bolam, Avtar Sur, Philip Mitchell, Jonathan Hutt, Nemandra A. Sandiford

**Affiliations:** 1 Trauma & Orthopaedics, St George’s Hospital, London, UK; 2 Joint Reconstruction Unit, Department of Orthopaedics, Southland Hospital, Invercargill, 9812 New Zealand; 3 Department of Surgery, Faculty of Medical and Health Sciences, University of Auckland, Auckland, New Zealand; 4 Department of Surgical Sciences, Division of Health Sciences, University of Otago, Dunedin, New Zealand

**Keywords:** Function, Satisfaction, Lipoedema, Lymphoedema, Total knee arthroplasty

## Abstract

**Background:**

The aim of this study was to define outcomes after total knee arthroplasty (TKA) in lymphoedema and lipoedema patients managed by a multidisciplinary team and daily compression bandaging.

**Methods:**

A retrospective study was performed in a single centre. Between 2007 and 2018, 36 TKA procedures were performed on 28 consecutive patients with a diagnosis of lymphoedema and lipoedema. Oxford Knee Scores (OKS), EuroQol-5D (EQ-5D) scores, satisfaction scores, radiographs, and complications were obtained at the final follow-up. Patients were admitted to the hospital up to two weeks prior to surgery and remained on the ward for daily compression bandaging by the specialist lymphoedema team.

**Results:**

Over the study period, 36 TKAs were performed on 28 patients (5 males, 23 females) with a mean age of 71 years (range 54–90). Of these, 30 TKAs were in patients with lymphoedema, five with lipoedema, and one with a dual diagnosis. Overall, 28 TKAs (21 patients) were available at the final follow-up with a mean follow-up time of 61 months (range 9–138). The mean BMI was 38.5 kg/m^2^. The mean pre-operative and post-operative Oxford Knee Score increased from 18 (range 2–38) to 29 (range 10–54); *p* < 0.001. EQ-5D score increased from 0.48 (range 0.15–0.80) to 0.74 (0.34–1.00) (*p* < 0.001). Mean post-operative satisfaction was 7.6/10 (range 2–10), with 89.3% TKAs satisfied. Complications were one (4%, 1/28) deep vein thrombosis, one superficial wound infection, one prosthetic joint infection, one stiff knee requiring manipulation, and one intra-operative femoral fracture.

**Conclusions:**

Lymphoedema and lipoedema should not be seen as barriers to TKA if adopting a multidisciplinary approach.

## Introduction

Total knee arthroplasty (TKA) is being performed on patients with an increasing number of co-morbidities [1]. Outcomes in patients with significant co-morbidities, particularly high body mass index (BMI), have been shown to be inferior to healthier patients [2]. Lymphoedema is increasingly being recognised in association with a high BMI, but a relative paucity of data exists on outcomes of TKA in this group.

Lymphoedema is characterised by limb oedema, fibrosis, and deposition of adipose tissue as a result of insufficient lymphatic drainage. The aetiology can be either primary or secondary. Primary lymphoedema occurs with genetic or developmental abnormalities of the lymphatic system. Secondary lymphoedema can be due to trauma, inflammation, malignancy, and iatrogenic post-surgery [3]. Patients with lymphoedema can present with recurrent cellulitis, lymphangitis, and ulceration, which increases the risk of superficial and deep wound infection in TKA [4].

Lipoedema is a separate clinical entity only occurring in women, frequently in the presence of morbid obesity, and is suspected to be oestrogen driven. Whilst the pathophysiology is poorly understood, the phenotype is well characterised by excess adipose tissue in the lower half of the body, sparing the feet, waist, chest, and shoulders. Other symptoms include leg pain, swelling, bruising, light touch sensitivity, and joint laxity [5]. Both lipoedema and lymphoedema exhibit exaggerated, chronic limb oedema that can pose significant technical challenges for arthroplasty surgeons.

Due to the higher risk of both surgical and medical complications, TKA in patients with raised BMI and lymphoedema is associated with an increased cost [6]. There has been a focus on quantifying risks in this patient group; however, there is a relative lack of data on clinical outcomes and patient satisfaction. There is even less data available on patients with lipoedema.

The purpose of this study is to present the clinical and functional outcomes of patients with lymphoedema and lipoedema using a standardised, multidisciplinary protocol at a mean five year follow-up.

## Methods

A retrospective study was performed. All patients with a diagnosis of lymphoedema and lipoedema and treated with a primary TKA in our institution between 2007 and 2018 were included. Patient demographics and classification by the American Society of Anaesthesiology Scale (ASA) [7] and the Charlson Co-Morbidity Index (CCI) [8] were recorded.

All patients were managed by a multidisciplinary team, including specialists in lymphoedema treatment. Patients identified for surgery were discussed with the lymphoedema team pre-operatively. Patients were admitted to the hospital up to two weeks before their surgery for daily compression bandaging of both limbs performed by the lymphoedema specialist nurses to reduce the volume of oedema and bulk of soft tissue around the knee. Patients were encouraged to maintain their mobility during this period. This compression bandaging was continued up to one week post-operatively with physiotherapy. Patients were advised to wear their full-length, above-knee compression stockings upon discharge.

The pre-operative and post-operative patient function was assessed using the Oxford Knee Score (OKS) [9] and health status using the EuroQol-5D (EQ-5D) score [10]. Patients were then asked to describe their overall satisfaction at the final follow-up, using a Likert scale of 0–10, with 0 being least happy and 10 most happy. All complications were recorded, including infection and venous thromboembolism.

The Press Fit Condylar prosthesis (DePuy Synthes, Warsaw, IN) was used in all cases. All operations were performed by a consultant orthopaedic surgeon experienced in TKA. Prophylactic antibiotics were given at induction of surgery and continued for 24 h post-surgery. Tranexamic acid was administered intravenously prior to the skin incision. Tourniquets were not used. All patients received thromboprophylaxis according to guidelines provided by the National Institute for Health and Clinical Excellence [11].

All radiographs were reviewed by at least two authors and assessed for signs of implant loosening, including radiolucent lines and any change in the position of the femoral and tibial components.

Data were tabulated using Microsoft Excel, version 16.5 (Microsoft Corporation, Redmond, WA). Statistical analysis was performed using the Student’s *t*-test. The level of statistical significance was set at *p* < 0.05.

## Results

### Patient demographics and functional outcomes

Between 2007 and 2018, 36 TKAs were performed on 28 patients (5 males, 23 females). Five TKAs were performed in patients with lipoedema and one TKA in a patient with a dual diagnosis of both lymphoedema and lipoedema (Table 1). At the final follow-up, four patients died due to causes unrelated to their surgery. Of these, three died more than seven years following their surgery. The remaining patient died two years post-operatively from sepsis secondary to an abdominal source. One patient declined to participate, and two patients could not be contacted despite multiple attempts. Contact with their general practitioners revealed that they had not presented with issues related to their operated knees. Loss to follow-up was 22%, with 28 TKAs (21 patients) available at the final follow-up (Table 1). All results are presented, excluding those lost to follow-up.

**Table 1:** Inclusion and loss to follow-up. ‘Dual diagnosis’, both lymphoedema and lipoedema; *TKA*, total knee arthroplasty

	Total TKR	Patients	Bilateral TKA patients	Lymphoedema TKA	Lipoedema TKA	‘Dual diagnosis’ TKA
Intention to treat	36 (100%)	28	8	30	5	1
Loss to follow-up	8 (22%)	7	1	8	0	0
Final follow-up	28 (78%)	21	7	22	5	1

The mean age was 71 years (range 54–90 years). The mean ASA grade was 2.9 (range 2–3). The mean body mass index (BMI) was 38.5 kg/m^2^. The mean CCI was 4.2 (range 1–10). A full list of patient co-morbidities and their frequency is presented in Table 2. The underlying diagnosis in all cases requiring TKA was symptomatic osteoarthritis. The mean follow-up was 61 months (range 7–138 months). Patients stayed on average 21 days in the hospital (range 3–50). This included the entire period of banding and the TKA procedure. One patient with lipoedema declined daily compression bandaging due to concerns regarding frail and thin skin on her leg. She had staged bilateral TKA procedures and remained in hospital for three and four days following her right and left TKA procedures respectfully. She had no wound-related complications.

**Table 2:** Frequency of co-morbidities

Co-morbidity	Frequency *n* (% of TKAs)
Asthma	4 (14%)
Atrial fibrillation on anticoagulant	3 (11%)
Bronchiectasis	1 (4%)
Congestive cardiac failure	6 (21%)
Chronic obstructive pulmonary disease (COPD)	1 (4%)
Cerebrovascular accident (CVA)	1 (4%)
Deep vein thrombosis (DVT)	1 (4%)
Depression	1 (4%)
Hypertension	10 (11%)
Ischaemic heart disease (IHD)	1 (4%)
Malignancy	5 (18%)
Obstructive sleep apnoea (OSA)	2 (7%)
Psoriasis	1 (4%)
Rheumatoid arthritis	3 (11%)
Type 2 diabetes mellitus	10 (36%)

The mean OKS increased from 18 (range 2–38) to 29 (range 10–54); *p* < 0.001. The mean total EQ-5D pre-operatively was 0.48 (range 0.15–0.80). The mean total EQ-5D post-operatively was 0.74 (range 0.34–1.00) (*p* < 0.001). The greatest change in EQ-5D was seen in the pain and discomfort domains, which improved by 0.10 points (equivalent to an improvement from “severe” to “slight” pain) (Table 3). The mean overall satisfaction score was 7.6/10 (range 2–10). Three TKAs (10.7%) scored less than 5/10 and were considered dissatisfied. Six TKAs were performed in patients with lipoedema. The mean CCI in this group was 2.4 (range 2–-3). For patients with lipoedema, t mean OKS was 11 (range 2–30) pre-operatively and 36 (range 25–34) post-operatively (*p* < 0.001). The EQ-5D was 0.40 (range 0.15–0.70) pre-operatively and 0.85 (range 0.62–1.00) post-operatively (*p* < 0.003).

**Table 3:** EQ-5D domains. Each qualitative category (i.e. unbearable; severe; moderate; slight; none) is separated by 0.05 points. An increase in 0.05 points is equivalent to an improvement from severe to moderate, or from moderate to slight

	EQ-5D domains				
	Mobility	Pain & discomfort	Self-care: washing & dressing	Work & leisure	Anxiety & depression
Pre-operative (mean)	0.07	0.04	0.11	0.08	0.14
Post-operative (mean)	0.13	0.14	0.16	0.13	0.13
Change in score	0.07	0.10	0.05	0.05	0.04

### Radiographic outcomes

Standardised anteroposterior and lateral radiographs were obtained pre-operatively and at each follow-up visit. These were assessed by 2 reviewers on each occasion. No progressive radiolucent lines were encountered on either the femoral or tibial component up to the time of final follow-up. No change in component position was observed when compared to previous imaging.

### Complications

One (1/28, 4%) deep vein thrombosis occurred post-operatively in a patient with no previous history of venous thromboembolism and was treated with a course of rivaroxaban. One TKA required a manipulation under anaesthetic for knee stiffness and reduced range of motion. One case of prosthetic joint infection (PJI) occurred. This patient had been symptomatic with new-onset back pain pre-operatively and was diagnosed with multiple myeloma involving her spine six weeks post-operatively. The patient received chemotherapy immediately after diagnosis. PJI was diagnosed with a positive joint aspirate during chemotherapy, and the patient received six weeks of intravenous antibiotics followed by six weeks of oral antibiotics. No further surgical procedures were performed. One TKR in a patient with lipoedema required two weeks of post-operative antibiotics for a superficial wound infection post-operatively. There were no reoperations for haematoma evacuation and no instances of wound breakdown requiring readmission. One non-displaced femoral periprosthetic fracture occurred intraoperatively. This was recognised on post-operative radiographs. The patient’s BMI was 55 kg/m^2^. A decision was made to treat this non-operatively with protected weight-bearing for six weeks at which point there was evidence of clinical and radiologic union, and she was allowed to weight bear. There were no revisions or impending revisions at the time of the final follow-up. All patients were managed post-operatively in the orthopaedic ward; no patients required intensive care unit admission following their surgery.

## Discussion

Lymphoedema is estimated to affect 1 in 1000 patients in the USA [12] and 140–250 million people worldwide [3]. Obesity is closely linked to lymphoedema. The risk of developing post-surgical lymphoedema is threefold in patients with a BMI greater than 30 kg/m^2^ [13]. Lipoedema is estimated to affect 11% of women in Germany. On microscopy, the excess fat in lipoedema shows diffuse peri-lymphatic inflammatory infiltrate and fibrosis that is not present normally. The excess fat is also metabolically inaccessible to diet or exercise [5]. The two conditions are distinct: the lymphatic system has been shown to be essentially normal in cases of lipoedema assessed by lymphoscintigraphy or lymphangiography [14]. However, both conditions are primarily treated by decongestive compression bandaging and surgical resection as second-line therapy if this fails. Increased BMI and circumference of the limb pose difficulty for surgical exposure and intraoperative visualisation resulting in surgical times up to 23% longer in patients with BMI 35–40 kg/m^2^ compared to those with normal BMI [15]. This can lead to delayed wound healing, venous thromboembolism (VTE), and infection [, 4, 16]. The functional outcome of TKA on patients with higher BMI has been encouraging [17].

However, less is known about clinical outcomes and patient satisfaction after TKA in lymphoedema. To our knowledge, this is the first study to report outcomes in patients with a diagnosis of lipoedema. It is also the first to describe the results of multidisciplinary management of patients with lymphoedema and lipoedema requiring TKA. Our institution has a dedicated lymphoedema management team who are closely involved with the management of every patient undergoing surgery.

The results of this study suggest that patients with lymphoedema and lipoedema have significant improvement in their functional outcomes up to five years following TKA. Shrader and Morrey [4] reported improved Knee Society scores from a mean of 47 pre-operatively to 87 post-operatively (*p* < 0.0001), and functional knee scores from a mean of 36 to 59, respectively. The mean BMI in their cohort of 83 TKAs was 33.9 kg/m^2^. Napier et al. [18] reviewed 49 morbidly obese patients (BMI range 40–50 kg/m^2^, mean 41.8) and found that one year post-operative OKS improved by a mean of 22.7 kg/m^2^. This was not statistically different to 50 paired non-obese patients (BMI < 30 kg/m^2^), who also improved by 20.5 points at one year. This magnitude of improvement in OKS can be sustained at a five year follow-up, with improvements of 25.3 and 21.1 points in cemented and uncemented TKA, respectively [19]. The OKS in our cohort improved by a mean of 11 points at a mean follow-up of 61 months (5.1 years). However, patients still reported significantly improved overall EQ-5D and improved pain and discomfort scores, suggesting TKA in lymphoedema remains clinically beneficial in this group of patients. Patient satisfaction following TKA in the general population has been reported to be 70–84% of patients [20], with some studies suggesting as high as 95–98%, even in the presence of morbid obesity [19, 21]. The rate of satisfaction with TKA in patients with lymphoedema and lipoedema in this study is 89.3%. This is comparable to the most encouraging results in both the general population and patients with obesity. Three TKAs (10.7%) rated their satisfaction as less than 5/10 points. These patients were considered dissatisfied.

The mean ASA grade in our population was 2.9, and the mean CCI was 4.2, with 21% of patients suffering from congestive cardiac failure and 36% with type 2 diabetes mellitus. This reflects a population with higher than average co-morbidities in the UK, where 67% of patients are ASA 2 [22]. The frequency of VTE complicating TKA in the general population has been reported to be 1%, rising to 2% in obese patients and 3.1% in morbidly obese patients [21]. Less is known about the incidence of symptomatic VTE in patients with lymphoedema. Morrey and Shrader [4] reported this to be 3.6%: to the author’s knowledge, this is the only report. One case of symptomatic VTE was encountered in our cohort (3.8%): a deep venous thrombosis (DVT) without pulmonary emboli.

The incidence of PJI following TKA has also been reported to increase with rising BMI. In the general population, the risk is 0.7%, rising to 1.6% in obese patients and 3.4% in morbidly obese patients. Revision TKA has an associated incidence of PJI of 5.5% [22]. The risk of superficial and deep infection following primary TKA in patients with lymphoedema has been reported to be 12% and 7% [4]. This supports findings that lymphoedema is potentially the strongest predictor of treatment failure after a two-stage revision for infected TKA [23]. We encountered one case of both superficial (3.8%) and deep infection (3.8%). The single case of deep PJI in this study occurred in the context of immunosuppression, multiple myeloma, and chemotherapy only six weeks post-operatively. We experienced one case of intra-operative femur fracture (3.8%). This is similar to the report by Shrader and Morrey [4]. We also did not encounter some of the other complications presented in their report, including post-operative flexion contracture, arterial thrombosis, or post-operative haematoma.

This study demonstrates a low infection rate and positive functional and clinical outcomes but is limited by a retrospective design and small numbers. However, the volume of lymphoedema and lipoedema patients undergoing TKA is limited. Larger, multi-centre studies with a control group are required to confirm whether multidisciplinary management and compression bandaging has an effect beyond routine best practice management.

In a multidisciplinary setting, with pre- and post-operative compression bandaging, lymphoedema patients are satisfied with TKA and report improved outcomes. In this cohort, low rates of infection were observed compared with the only previously published study in lymphoedema. We suggest that with this structured approach, lymphoedema and lipoedema should not be seen as a barrier to TKA. Peri-operative lymphoedema treatment can be provided to patients undergoing TKA in a safe, effective, and low-cost manner through a multidisciplinary approach.

## Data Availability

This manuscript has associated data in a data repository that is available on request from the corresponding author.

## Abbreviations

ASA: American Society of Anaesthesiology Scale
BMI: Body mass index
CCI: Charlson Co-Morbidity Index
EQ-5D: EuroQol-5D
OKS: Oxford Knee Score
PJI: Prosthetic joint infection
TKA: Total knee arthroplasty
VTE: Venous thromboembolism
